# Supplementary material for: Thermophilic Inorganic Pyrophosphatase Ton1914 from Thermococcus onnurineus NA1 Removes the Inhibitory Effect of Pyrophosphate
Source: Int J Mol Sci. 2022 Oct 22;23(21):12735. doi: 10.3390/ijms232112735 (PMC9653972; doi:10.3390/ijms232112735)
Supplement: Supplementary file 1 [file ijms-23-12735-s001.zip › ijms-1921315-supplementary.pdf]

Supplementary Materials

Thermophilic Inorganic Pyrophosphatase Ton1914 from *Thermococcus onnurineus* NA1 Removes the Inhibitory Effect of Pyrophosphate

Yajing Li, Xue Yang and Renjun Gao *

Key Laboratory for Molecular Enzymology and Engineering of Ministry of Education, School of Life Science, Jilin University, Changchun 130021, China
* Correspondence: gaorj@jlu.edu.cn; Tel.: +86-186-0431-3058

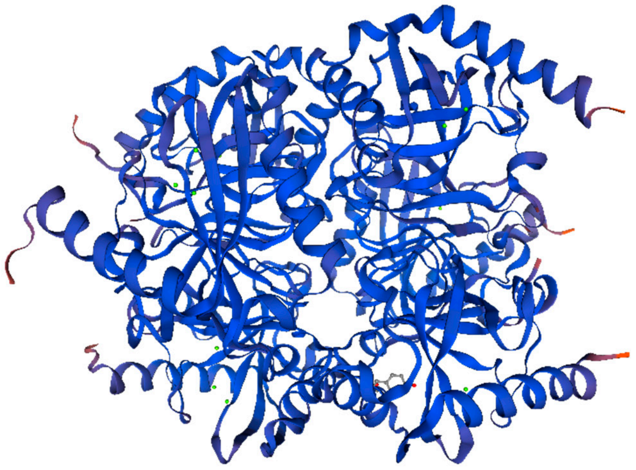

Figure S1. Structural information of Ton1914 modeled by Discovery Studio. The template selected is Tt-IPPase from Thermococcus thioreducens and sequence identity is 96.07 %.

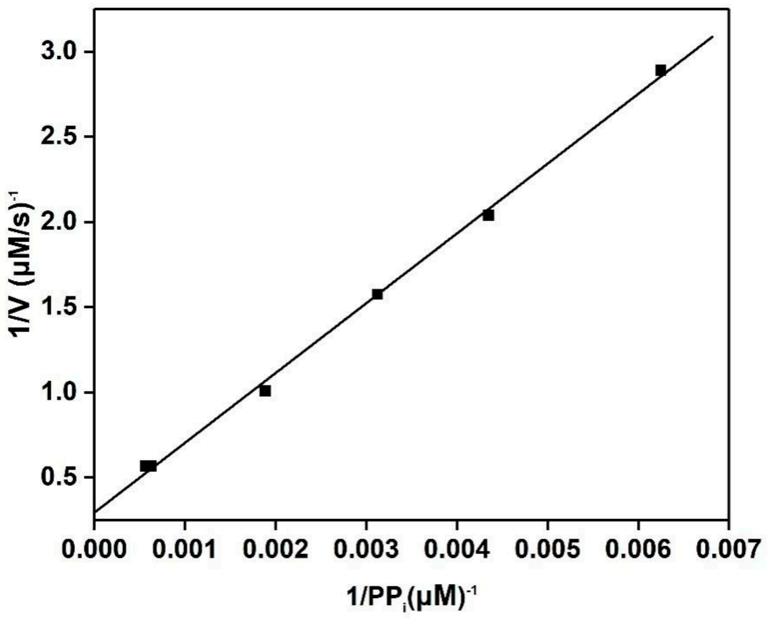

Figure S2. The kinetic curves of Ton1914. The experiments were conducted at 80°C with Tris-HCl buffer (pH 9.0) for 9000 s.

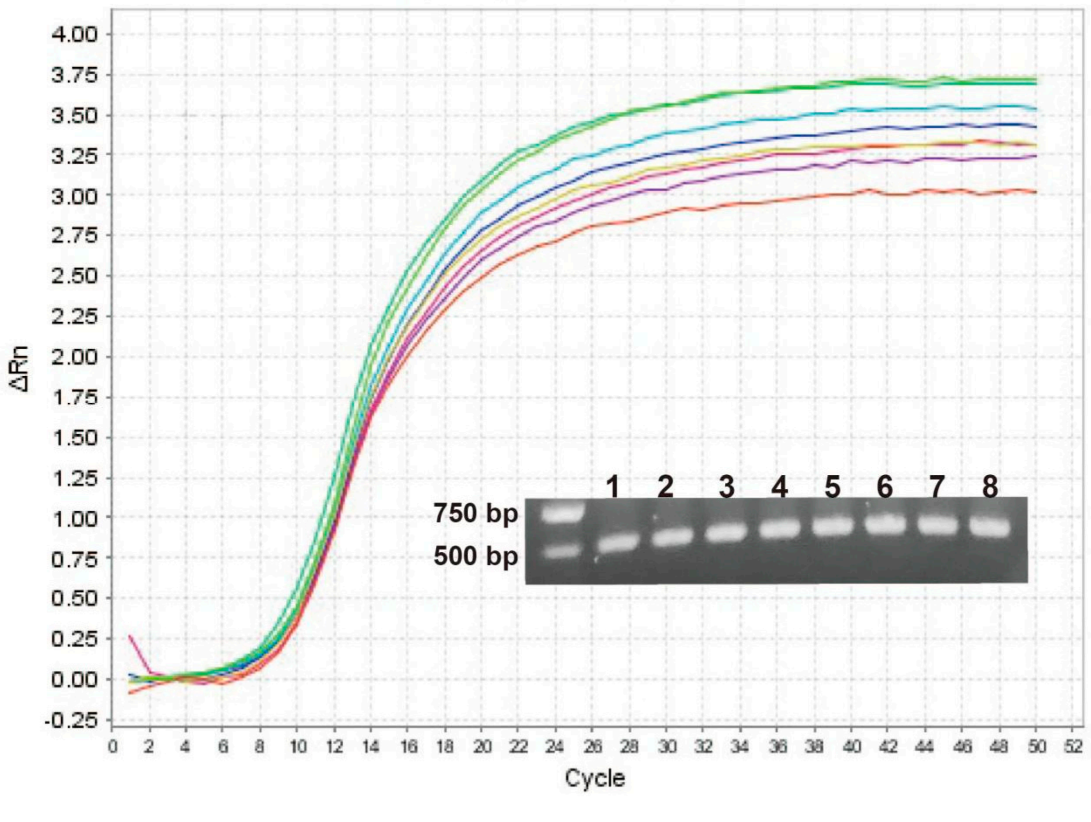

Figure S3. Optimal addition of Ton1914 in RT-PCR reactions a. The concentrations of Ton1914 added to each line in the graph from top to bottom were 0.0007 mg/mL, 0.0005

mg/mL, 0.0009 mg/mL, 0.0011 mg/mL, 0.0003 mg/mL, 0.0013 mg/mL, 0.0015 mg/mL and 0 mg/mL, respectively； 1-8 in inset b are 0 mg/mL, 0.0003 mg/mL, 0.0005 mg/mL, 0.0007 mg/mL, 0.0009 mg/mL, and 0.0011 mg/mL, 0.0013 mg/mL and 0.0015 mg/mL.respectively. Perform qPCR with Tfu2902 template.

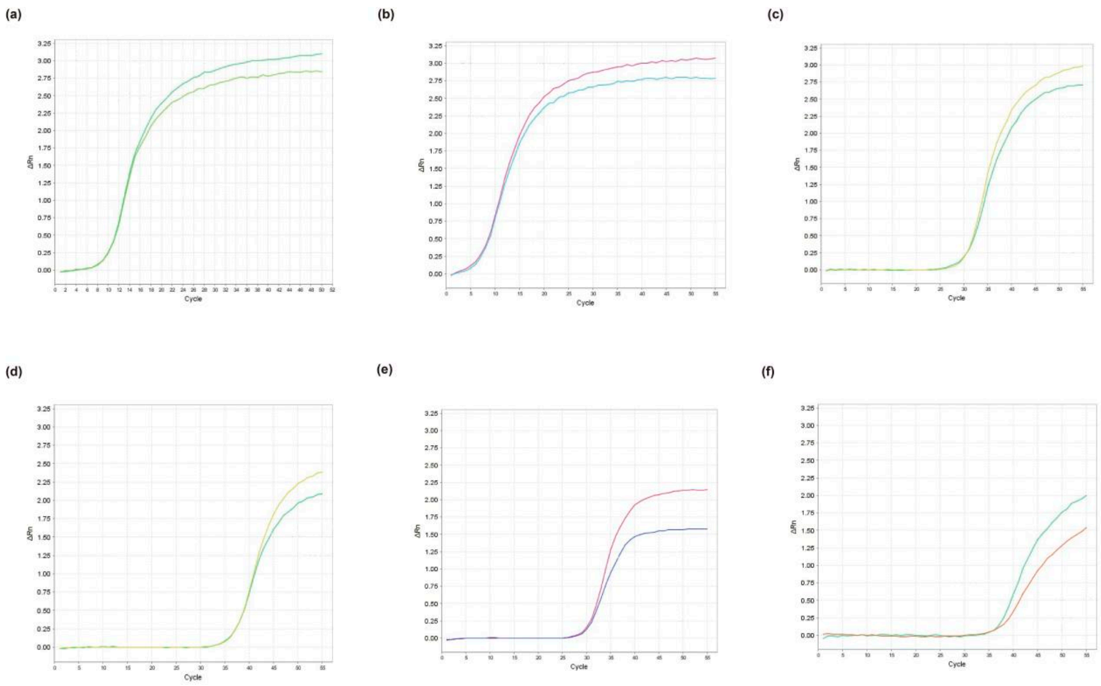

Figure S4. qPCR promotion effect of Ton1914 on DNA of different sizes. a, Tfu2902; b, Gs01315; c, Aaci0783; d, Tl08779; e, Cus2552; and f, pET28a–*Tn0602*. The upper curve is adding Ton1914 group, the lower curve is not adding Ton1914 group.
